# Visual fixation in the vegetative state: an observational case series PET study

**DOI:** 10.1186/1471-2377-10-35

**Published:** 2010-05-26

**Authors:** Marie-Aurélie Bruno, Audrey Vanhaudenhuyse, Caroline Schnakers, Mélanie Boly, Olivia Gosseries, Athena Demertzi, Steve Majerus, Gustave Moonen, Roland Hustinx, Steven Laureys

**Affiliations:** 1 Coma Science Group and Cyclotron Research Centre, University of Liège, Liège, Belgium; 2 Department of Neurology, University Hospital of Liège, Liège, Belgium; 3 Research Center for Cognitive and Behavioral Neuroscience, University of Liège, Liège, Belgium; 4 Department of Nuclear Medicine, University Hospital of Liège, Liège, Belgium

## Abstract

**Background:**

Assessment of visual fixation is commonly used in the clinical examination of patients with disorders of consciousness. However, different international guidelines seem to disagree whether fixation is compatible with the diagnosis of the vegetative state (i.e., represents "automatic" subcortical processing) or is a sufficient sign of consciousness and higher order cortical processing.

**Methods:**

We here studied cerebral metabolism in ten patients with chronic post-anoxic encephalopathy and 39 age-matched healthy controls. Five patients were in a vegetative state (without fixation) and five presented visual fixation but otherwise showed all criteria typical of the vegetative state. Patients were matched for age, etiology and time since insult and were followed by repeated Coma Recovery Scale-Revised (CRS-R) assessments for at least 1 year. Sustained visual fixation was considered as present when the eyes refixated a moving target for more than 2 seconds as defined by CRS-R criteria.

**Results:**

Patients without fixation showed metabolic dysfunction in a widespread fronto-parietal cortical network (with only sparing of the brainstem and cerebellum) which was not different from the brain function seen in patients with visual fixation. Cortico-cortical functional connectivity with visual cortex showed no difference between both patient groups. Recovery rates did not differ between patients without or with fixation (none of the patients showed good outcome).

**Conclusions:**

Our findings suggest that sustained visual fixation in (non-traumatic) disorders of consciousness does not necessarily reflect consciousness and higher order cortical brain function.

## Background

It is still a matter of debate whether visual fixation indicates "automatic" subcortical processing (i.e., is compatible with the diagnosis of the vegetative state; VS [1,2]) or whether it is a cognitively mediated behavior that heralds consciousness and higher order cortical processing (i.e., sufficient for the diagnosis of the minimallyconscious state; MCS [3]). According to the Multi Society Task Force on PVS in "rare cases, patients who have no other evidence of consciousness over a period of months to years have some degree of briefly sustained visual fixation, which is believed to be mediated through brainstem structures"[1]. Similarly, UK guidelines state that "visual fixation of a target" is a "compatible but atypical feature" of the VS [2]. The Aspen Neurobehavioral Conference [3], however, considered that "sustained fixation that occurs in direct response to moving or salient stimuli" is a clinical criterion defining MCS. We here compared cerebral metabolism of VS patients (of whom none showed visual fixation) with patients showing sustained (i.e., >2 s.) visual fixation but whose clinical features were in all other aspects typical of VS. To the best of our knowledge, this study is the first to employ functional neuroimaging to document the neural correlate of ambiguous behavioral signs of consciousness in the challenging patients surviving an acute severe brain damage.

## Methods

Cerebral metabolic rates for glucose (CMRGlu) [4] were studied by means of [^18^F]-fluorodeoxyglucose-PET (FDG-PET) in ten patients with chronic (>4 weeks) post-anoxic encephalopathy (3 women, aged 46 ± 11 years) and 39 age-matched healthy controls (21 women; aged 45 ± 16 years). Patients were assessed by means of the Coma Recovery Scale-Revised (CRS-R) [5] and showed the clinical criteria of VS as defined by the Multi Society Task Force on PVS [1]: (i) no evidence of awareness of self or environment and an inability to interact with others; (ii) no evidence of sustained, reproducible, purposeful, or voluntary behavioral responses to visual, auditory, tactile, or noxious stimuli; (iii) no evidence of language comprehension or expression; (iv) intermittent wakefulness manifested by the presence of sleep-wake cycles; (v) sufficiently preserved hypothalamic and brain-stem autonomic functions to permit survival with medical and nursing care; (vi) bowel and bladder incontinence; and (vii) variably preserved cranial-nerve reflexes (pupillary, oculocephalic, corneal, vestibulo-ocular, and gag) and spinal reflexes. Patients' enrollment started February 2006 and ended July 2009. Five patients did not show visual fixation and five patients did - both groups were matched for age, etiology (all anoxic), time since insult and other clinical features (as illustrated by the CRS-R subscores shown in table 1).

**Table 1:** Demographic and clinical data of patients in vegetative state (VS; without visual fixation) and with visual fixation but otherwise showing all clinical features of the vegetative state.

	VS1	VS2	VS3	VS4	VS5	Fixation 1	Fixation 2	Fixation 3	Fixation 4	Fixation5
Gender (age, years)	62 (M)	35(F)	56(F)	53(M)	54(M)	51(M)	49(M)	26(M)	38(F)	37(M)
Etiology	Anoxic	Anoxic	Anoxic	Anoxic	Anoxic	Anoxic	Anoxic	Anoxic	Anoxic	Anoxic
Interval after insult	8 months	2 years	1.5 months	1 year	5 months	2 months	2 years	3 years	6 months	2 months
Auditory function*	Startle reflex	Startle reflex	Startle reflex	Startle reflex	Startle reflex	Localization of sounds	Startle reflex	Startle reflex	Startle reflex	Startle reflex
Visual function*	None	Blink to threat	None	Blink to threat	None	Visual fixation	Visual fixation	Visual fixation	Visual fixation	Visual fixation
Motor function*	Flexion to pain	Flexion to pain	Flexion to pain	Abnormal posturing	Abnormal posturing	Flexion to pain	None	Abnormal posturing	Flexion to pain	Abnormal posturing
Oromotor/Verbal function*	Oral reflexes	Oral reflexes	Oral reflexes	Oral reflexes	Oral reflexes	None	Oral	Oral	Oral	Oral
							reflexes	reflexes	reflexes	reflexes
Communication*	None	None	None	None	None	None	None	None	None	None
Arousal*	Without stimulation	Without stimulation	With stimulation	Without stimulation	With stimulation	Without stimulation	With stimulation	Without stimulation	With stimulation	Without stimulation
Outcome after 1 year	No command following*	No command following*	No command following*	No command following*	Death	Death	No command following*	No command following*	Death	No command following*

* as assessed by Coma Recovery Scale-Revised [5]

Visual fixation was assessed as defined in the CRS-R [5]: a brightly colored object was presented 6 to 8 inches in front of the patient's face and was rapidly moved to upper, lower, right and left visual fields for a total of 4 trials. Fixation was considered as present when the eyes changed from the initial fixation point and refixated on the new target location for more than 2 seconds (at least 2 episodes of fixation were required). Each patient was assessed in the sitting position and patient preparation employed a standardized arousal facilitation protocol [5]. The goal of this intervention was to prolong the length of time the patient maintained arousal. All patients were assessed free of sedative drugs.

FDG-PET data were pre-processed [4] and analyzed using Statistical Parametric Mapping (SPM8; http://www.fil.ion.ucl.ac.uk/spm). We looked for brain regions where CMRGlu was different between: (i) patients without fixation and healthy controls (ii) patients with fixation and healthy controls (iii) patients without fixation as compared to patients with fixation. In a second step, we used a psychophysiological interaction analysis [6] to test for differences in functional cortico-cortical connectivity (employing the same methodology as we have previously published [4]) in patients without and with visual fixation. The design matrix included the same scans as in the first analysis and took into account group differences in mean levels of glucose consumption. Now we looked for cortical regions that experienced a significant difference in reciprocal modulation with/from the visual cortical regions (V1 and V2). Seed region of interest (Brodmann area's 17 and 18) was taken from previously published probabilistic standardized 3D structural volumes of V1 and V2 [7]). Results were thresholded for significance at whole-brain false discovery rate corrected p < 0.05. Patients were prospectively followed for at least 12 months by means of repeated CRS-R testing. Good outcome was defined as recovery of functional communication. The study was approved by the Ethics Committee of the Faculty of Medicine of the University of Liege. Written informed consent was obtained from healthy controls and patients' legal representative.

## Results and Discussion

**Figure 1:**
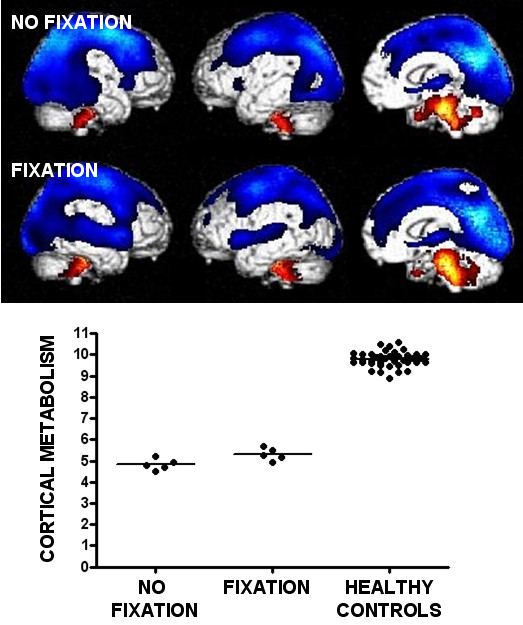
**Brain areas with impaired cerebral glucose metabolism (blue) in patients in a vegetative state (without fixation) and in patients with visual fixation but showing all other clinical features of the vegetative state**. Brainstem and cerebellum show a relatively preserved brain function (red). The graph illustrates the decreased metabolic activity (expressed in arbitrary units as normalized [^18^F]-fluorodeoxyglucose neuronal uptake) in the fronto-parietal cortical network in both patient groups as compared to normal healthy controls.

When compared to healthy participants, VS patients without fixation showed metabolic dysfunction in a widespread cerebral network encompassing bilateral thalami and fronto-temporo-parietal associative cortices. Areas that were relatively spared were confined to the brainstem and cerebellum. Patients with visual fixation but otherwise showing clinical characteristics typical of VS showed a similar pattern of metabolic dysfunction (when compared to the control group; see Figure 1 and table 2). The direct comparison between both patient groups (without and with fixation) did not show any differences in cerebral metabolism. We observed no difference in metabolism in visual areas (V1 and V2) and no difference in cortico-cortical connectivity with these visual areas between patients without and with visual fixation. The follow-up study showed no differences in outcome between both groups at 12 months follow-up (Table 1).

**Table 2:** Peak voxels of areas showing lower metabolism in patients as compared to controls (coordinates are in standardized stereotaxic Montreal Neurological Institute space).

Peak voxel of cluster	*x *(mm)	*y *(mm)	*z *(mm)	z-value	Correcte p-value
**A**. PATIENTS WITHOUT FIXATION					
PCC/precuneus	8	-48	32	Inf	< 0.0001
ACC/mesiofrontal	4	16	32	4.01	< 0.0001
L posterior parietal	-42	-74	42	5.66	< 0.0001
R posterior parietal	34	-66	58	6.28	< 0.0001
L dorsolateral prefrontal	-48	-22	56	5.60	< 0.0001
R dorsolateral prefrontal	46	4	58	7.01	< 0.0001
L thalamus	-6	-14	6	5.07	< 0.0001
R thalamus	10	-18	8	5.64	< 0.0001
**B**. PATIENTS WITH FIXATION					
PCC/precuneus	-6	-56	26	Inf	< 0.0001
ACC/mesiofrontal	6	4	38	4.90	< 0.0001
L posterior parietal	-44	-58	56	5.24	< 0.0001
R posterior parietal	34	-85	34	5.74	< 0.0001
L dorsolateral prefrontal	-42	-4	58	5.72	< 0.0001
R dorsolateral prefrontal	48	4	58	6.85	< 0.0001
L thalamus	-6	-14	6	4.73	< 0.0001
R thalamus	12	-14	8	5.65	< 0.0001

R: right; L: left; PCC: posterior cingulate cortex; ACC: anterior cingulate cortex

## Conclusions

We here provide evidence that sustained visual fixation in patients otherwise showing the clinical criteria of VS is not accompanied by any difference in cortical metabolism when compared to "typical" VS patients lacking visual fixation. It should be stressed that our findings pertain to anoxic etiology (post-traumatic cases were excluded because the ensuing variability in focal brain damage makes spatial normalization of PET images problematic). We also point out the difficulty with non-significant findings in small cohort studies where the lack of difference may be the result of weak statistical power. However, the graphical illustration of the single-subject data of the functional segregation analysis shows an almost complete overlap between both patient groups and the functional integration analysis [6] shows comparable cortico-cortical connectivity with visual areas between patients without and with visual fixation. Our results in VS patients who did not show fixation are in line with previous studies demonstrating a widespread thalamocortical dysfunction in VS with only sparing of subcortical structures [e.g., see [4]]. The important finding here is that the presence of sustained visual fixation (here defined as at least 2 fixations of at least 2 seconds [5]) was not accompanied by any significant difference in cortical metabolism nor in cortico-cortical functional connectivity. It should be noted that at less conservative threshold the brainstem (coordinates x = 12 y = -36 z = -36 mm; Z value = 2.97; uncorrected p = 0.001) showed higher metabolism in patients with sustained visual fixation as compared to patients without fixation.

Our findings are in line with previous studies of "automatic" visual fixation of salient stimuli in blindsight [8], hemianopsia and visual agnosia [e.g., for review see [9]]. Similarly, recent functional neuroimaging studies in healthy subjects demonstrate that voluntary control of visual orienting eye movements are controlled by widespread dorsal frontoparietal networks [e.g., see [10,11]], shown to be dysfunctional in our reported patients with visual fixation. Finally, patients' one year follow up showed similar bad outcome in patients without and with visual fixation (none recovered command-following), in line with previous outcome data for anoxic VS [1].

In our view, the present results are of interest to clinical neurologists, who have taken visual fixation and tracking as being an important step in recovery of consciousness from the vegetative state [12]. The here presented novel approach of correlating specific behavioral signs in disorders of consciousness with functional neuroimaging results could help identifying their underlying functional neuroanatomy and possible reflection of conscious awareness [13]. Future studies should employ this methodology to increase our understanding of remaining ambiguous signs of consciousness such as for example the presence of orientation response to auditory stimuli or grimaces, abduction, flexion or orientation responses to noxious stimulation [14].

In conclusion, our findings suggest that in (non-traumatic) disorders of consciousness, sustained visual fixation is not accompanied by higher order frontoparietal integrative cortical brain function which is assumed to be associated with conscious awareness [15].

## Competing interests

The authors declare that they have no competing interests.

## Authors' contributions

MAB made substantial contributions to acquisition of data, analysis and interpretation of data as well as in drafting the manuscript. AV, CS and MB were implied in the interpretation of data as well as in drafting the manuscript; gave critical revision of the manuscript for important intellectual content. OG and AD were implied in collecting behavioural data. SM, RH and GM were involved in drafting the manuscript. SL made substantial contributions to conception and design and supervised this study. All authors read and approved the final manuscript.

## Pre-publication history

The pre-publication history for this paper can be accessed here:

http://www.biomedcentral.com/1471-2377/10/35/prepub
